# Increased Permeability of the Blood–Brain Barrier in a Diabetic Mouse Model (*Lepr^db^*^/*db*^ Mice)

**DOI:** 10.3390/ijms25147768

**Published:** 2024-07-16

**Authors:** Musaad A. Alshammari, Abdulaziz O. Alshehri, Faleh Alqahtani, Mohammad R. Khan, Muhammed A. Bakhrebah, Fawaz Alasmari, Tahani K. Alshammari, Shakir D. Alsharari

**Affiliations:** 1 Department of Pharmacology and Toxicology, College of Pharmacy, King Saud University, Riyadh 11495, Saudi Arabia; afaleh@ksu.edu.sa (F.A.); kmohammad@ksu.edu.sa (M.R.K.); ffalasmari@ksu.edu.sa (F.A.); talshammary@ksu.edu.sa (T.K.A.); sdalsharari@ksu.edu.sa (S.D.A.); 2 Department of Pharmacology and Toxicology (Graduate Student), Pharmacy College, King Saud University, Riyadh 11495, Saudi Arabia; aoshehri@outlook.com; 3 Life Science and Environment Research Institute, King Abdulaziz City for Science and Technology, Riyadh 11442, Saudi Arabia; mbakhrebah4@gmail.com

**Keywords:** *Lepr^db^*^/*db*^ mice, diabetes mellitus, blood–brain barrier, leptin receptor, obesity

## Abstract

Type 2 Diabetes Mellitus (T2DM) is linked to multiple complications, including cognitive impairment, and the prevalence of memory-related neurodegenerative diseases is higher in T2DM patients. One possible theory is the alteration of the microvascular and macrovascular environment of the blood–brain barrier (BBB). In this study, we employed different approaches, including RT-PCR, functional pharmacokinetic studies using sodium fluorescein (NaFL), and confocal microscopy, to characterize the functional and molecular integrity of the BBB in a T2DM animal model, leptin receptor-deficient mutant mice (*Lepr^db^*^/*db*^ mice). As a result, VCAM-1, ICAM-1, MMP-9, and S100b (BBB-related markers) dysregulation was observed in the *Lepr^db^*^/*db*^ animal model compared to littermate wild-type mice. The brain concentration of sodium fluorescein (NaFL) increased significantly in *Lepr^db/db^* untreated mice compared to insulin-treated mice. Therefore, the permeability of NaFL was higher in *Lepr^db^*^/*db*^ control mice than in all remaining groups. Identifying the factors that increase the BBB in *Lepr^db^*^/*db*^ mice will provide a better understanding of the BBB microvasculature and present previously undescribed findings of T2DM-related brain illnesses, filling knowledge gaps in this emerging field of research.

## 1. Introduction

Diabetes Mellitus (DM) is a heterogeneous metabolic disorder of carbohydrates, proteins, and fats. It is caused by reduced secretion of the insulin hormone produced by pancreatic beta cells [1,2,3,4]. It also refers to the deficiency of insulin or its resistance as a result of individual or environmental factors [5,6]. Factors that predispose individuals to DM include obesity, being overweight, physical inactivity, genes, and family history [3,7]. Over the years, the prevalence of DM worldwide has increased from 4.7% (108 million) in 1980 to 8.5% (422 million) in 2014 [8,9]. Notably, its prevalence has increased more in low- and middle-income countries than in high-income countries [10]. Mortality associated with the condition is also increasing. The mortality rate in 2016 was reported to be 5% higher than the rate in 2000 [9]. The International Diabetes Federation (IDF) originally estimated that the global incidence of DM will increase from 171 million in 2000 to 366 million by 2030 [11].

DM is a metabolic disease that is primarily characterized by hyperglycemia resulting from the insufficient release of insulin, a defect in insulin action, or both [3]. Physiologically, either β-cells are destroyed (i.e., Type 1 Diabetes Mellitus, T1DM) or insulin resistance occurs (i.e., Type 2 Diabetes Mellitus, T2DM) [12]. The chronic elevation of plasma sugar levels (hyperglycemia) has multiple consequences. The clinical manifestations can lead to the loss of function and the damage of tissues that will eventually cause the failure of organs, mainly in the eyes, kidneys, heart, and nerves [3,13].

The majority of diabetic patients fall into T2DM, which appears in almost 90–95% of diabetic patients [3]. The pathophysiology of T2DM starts from insulin resistance with relative insulin deficiency to an insulin secretion defect with insulin resistance [3]. The increase in the glucose level proportionally enhances the incidence of neuropathy disorders, stimulates reactive oxygen species (ROS) production, and increases inflammatory responses [13]. Additionally, it triggers the dysregulation of several pathways, such as polyol, hexosamine, protein kinase-C (PKC), and advanced glycation end products (AGE) pathways [14,15]. Hyperglycemia will lead to the disruption of endothelial cell integrity [16]. Chronic hyperglycemia is associated with macrovascular and microvascular illnesses such as retinopathy, nephropathy, and neuropathy.

On the level of microvascular injury, diabetes affects the capillary membrane by increasing the thickness, leading to various disorders such as hypertension and tissue hypoxia [13]. Additionally, a wealth of evidence has linked hyperglycemia to the pathogenesis of multiple neurological and psychiatric disorders such as ischemia, stroke, Alzheimer’s, schizophrenia, depression, and bipolar disorder. Most of them share a unifying feature: a disruption in the dynamics and structure of the BBB [17,18,19,20,21,22,23].

The blood–brain barrier (BBB) is a powerful selective barrier that controls the movement of molecules (such as oxygen, nutrients, and ions) between the blood and the brain. The BBB is a composite of endothelial cells, pericytes, and astrocytes at the cellular level [24]. In addition, tight junction proteins such as zonula occludin and claudins connect the junctions between endothelial cells. Together, the cellular structure and protein function of the BBB plays a crucial role in maintaining the integrity of the BBB and preventing the influx of any toxins or pathogens [25,26]. Neuroinflammation and oxidative stress are common causes of BBB disruption, resulting from their influence in decreasing the amount of tight junction proteins and increasing the permeability of the BBB [27]. All these changes resulting from neuroinflammation may cause different neurodegenerative diseases [28].

Multiple animal models have been utilized to study T2DM. One is leptin receptor mutant (*Lepr^db^*^/*db*^) mice [29]. Leptin is a hormone that passes to the central nervous system (CNS) and acts on the leptin receptor, stimulating functions related to hunger sensation, energy balance, and thermogenesis. It was linked to other physiological functions, including mood regulation, synaptic wiring, and neurogenesis [30]. *Lepr^db^*^/*db*^ mice harbor mutations in the leptin receptor, leading to dysfunctional signaling and recapitulating phenotypic behavioral and molecular features associated with DM and obesity [29,31].

Limited studies have addressed diabetes mellitus’ impact on the microvasculature of the CNS, specifically on the architecture of the BBB. In addition, functional, structural, and/or dynamic studies of BBB components are limited. Fundamental questions need to be addressed, such as: (1) What is the effect of long-term hyperglycemia on BBB function? (2) How does hyperglycemia impact the gene expression of BBB components? (3) What are the BBB transporting system’s dynamic changes and functions? (4) Is disruption of the BBB the leading cause of brain disorders, or is it an emerging symptom? What are the molecular links between brain disorders and the BBB? (5) Does the BBB participate in etiology or play a synergistic role in the disease’s symptoms?

Until now, many of these fundamental questions remain unanswered. Understanding the BBB’s microvascular and macrovascular elements underlying T2DM might be crucial for revealing the causation of many T2DM-related brain disorders and helping us find new pharmacological interventions. Here, our study focuses on a new functional role of the BBB in T2DM that could be a possible mechanism underlying reduced synaptic plasticity in the neuronal population in the *Lepr^db^*^/*db*^ mouse model and might answer some of these questions.

## 2. Results

### 2.1. The Molecular Expression of BBB Markers in the Brain Homogenate of Lepr^db/db^ Mice

**Figure 1:**
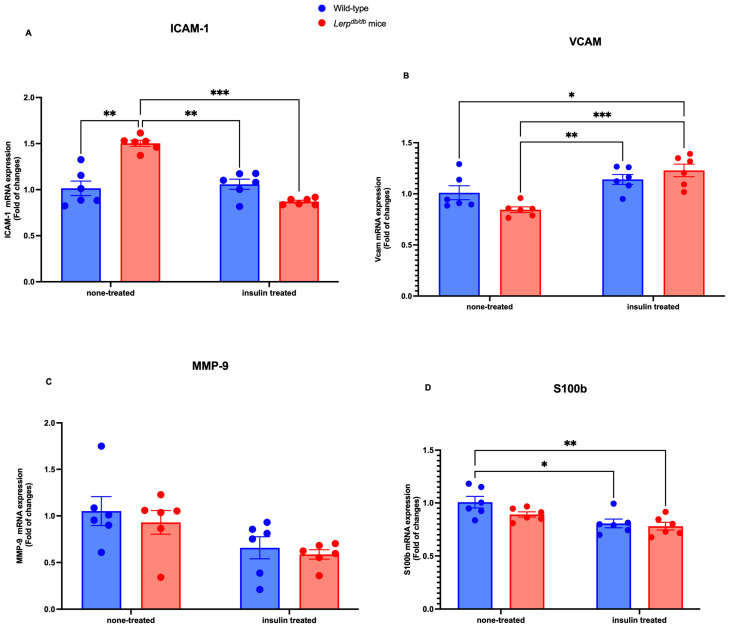
Comparison of ICAM-1, VCAM-1, MMP-9, and S100b mRNA expression in WT control and *Lepr^db^*^/*d*^ mice. Molecular examination of the effects of insulin on ICAM-1, VCAM-1, MMP-9, and S100b in the PFC homogenate. The mRNA expression level of ICAM-1 (**A**), VCAM-1 (**B**), MMP-9 (**C**), and S100b (**D**) was determined by quantitative RT-PCR with GAPDH as an internal control. Data were expressed as fold change and analyzed using a two-way ANOVA followed by a Tukey–Kramer post-test. Each value indicates the mean ± S.E.M. of three animals. The total number of biological and technical replicates is 6 per group. * *p* < 0.05; ** *p* < 0.01; *** *p* < 0.001. Groups are wild-type control = WT; wild-type treated with insulin = WT treated; *Lepr^db^*^/*db*^ = DB-CONT; and *Lepr^db^*^/*db*^ treated with insulin = DB + INS).

First, we examined the mRNA levels of the BBB-related markers ICAM-1, VCAM-1, MMP-9, and S100BS100b In the PFC homogenate, the level of ICAM-1 mRNA was significantly elevated in *Lepr^db^*^/*db*^ mice, compared with the other groups (*p* < 0.05) (Figure 1A). Conversely, the mRNA level of VCAM-1 was significantly reduced in *Lepr^db^*^/*db*^ mice (*p* < 0.01) and dramatically increased *in Lepr^db^*^/*db*^ mice treated with insulin (*p* < 0.001) (Figure 1B). Our analyses indicated that MMP-9 was not altered among the groups (Figure 1C). Our investigation was then extended to examine S100b. We found that the mRNA level of S100b was significantly reduced in *Lepr^db^*^/*db*^ insulin-treated mice (*p* < 0.001). In addition, the mRNA level of S100b was reduced in *Lepr^db^*^/*db*^ mice treated with insulin (*p* < 0.01) compared to the control mice. Similarly, it was reduced in control mice treated with insulin (*p* < 0.05) compared to the control mice (Figure 1D). Our quantitative RT-PCR analysis revealed that the integrity of the BBB was altered in tested groups.

### 2.2. Immunohistochemistry of ZO-1 in the Prefrontal Area of Lepr^db/db^ and Control Mice

**Figure 2:**
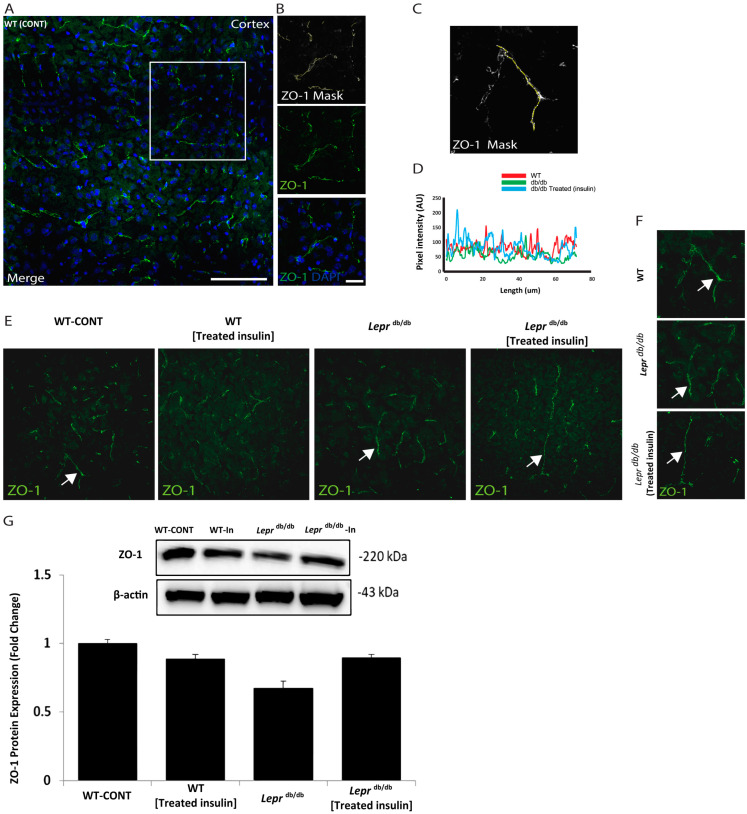
The expression pattern of ZO-1 in the cortical area of *Lepr^db^*^/*db*^ and control mice. Examination of the architecture of ZO-1 in the cortex slices of *Lepr^db^*^/*db*^ and control mice. (**A**) ZO-1 immunoreactivity (green) and DAPI indicate nuclear staining (blue). (**B**) Representative higher-resolution views of the white box of the ZO-1-positive segment (green) and the ZO-1 ROI mask were generated by adjusting the threshold of ZO-1 using ImageJ software in the cortex (ImageJ, U. S. National Institutes of Health, Bethesda, MD, USA, (http://imagej.nih.gov/ij) added in methodology). (**C**) High-power (top) mask and (**D**) the total quantifications of ZO-1 expression. (**E**) Representative immunostaining images of ZO-1 (green), arrows indicate ZO-1 fluorescent signals. (**F**) ZO-1 immunoreactivity (green). Right panels represent high magnification of the white arrows in (**E**). (**G**) Immunoblot detection of ZO-1 in whole mouse cortex homogenates from *Lepr^db^*^/*db*^, wild-type, *Lepr^db^*^/*db*^, and wild-type treated mice. Statistical differences were assessed using a two-way ANOVA followed by a Tukey–Kramer post-hoc test. Each value indicates the mean ± S.E.M. of 3 animals per group. Groups are Wild-type control = WT (lean); *Lepr^db^*^/*db*^ = *Lepr^db^*^/*db*^; and *Lepr^db^*^/*db*^ treated with Insulin = *Lepr^db^*^/*db*^ Insulin-Treated. Scale bars represent 100 μm in A and 20 μm in (**B**).

Next, we explored the architecture of the BBB in the cortex of the tested groups. We examined the expression pattern of ZO-1, a protein that links transmembrane components to the actin cytoskeleton (Figure 2). As illustrated in Figure 2, we used the mask to track the intensity and pattern of ZO-1 immunofluorescence. Then, we examined ZO-1 expression in the wild-type mice, *Lepr^db^*^/*db*^ mice, and *Lepr^db^*^/*db*^ mice treated with insulin (Figure 2). Notably, the expression intensity and pattern of ZO-1 were comparable among all groups (Figure 2). This indicates that treatment with insulin may have a profound molecular impact on ZO-1 expression. We further examined occludin levels (Supplementary Figure S1), and we found a lack of statistical significance.

### 2.3. BBB Permeability Analysis Using Na+ Fluorescein

**Figure 3:**
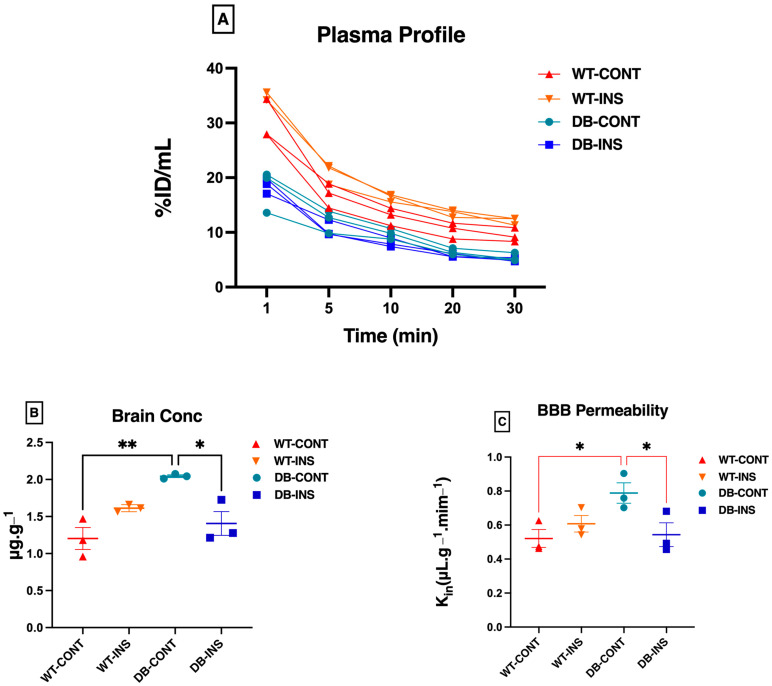
Permeability characteristics of the BBB using NaFL in *Lepr^db^*^/*db*^ and control mice. (**A**) The plasma concentration profile for sodium fluorescence (NaFL) from 0 to 30 min after intravenous bolus injection. (**B**) The brain concentration for sodium fluorescence (NaFL) at 30 min after intravenous bolus injection. (**C**) The clearance into the brain tissue for sodium fluorescence (NaFa) after intravenous bolus injection. * *p* < 0.05; ** *p* < 0.01. Data represent mean ± S.E.M.

The outcomes of the BBB permeability experiment are depicted in Figure 3. All Sodium Fluorescein (NaFL) concentrations measured from plasma and brain samples fell within the standard curve. The plasma concentration–time curves (%ID/mL) of the four groups are presented in Figure 3A. This shows that the plasma concentration profile decreased from 0–30 min. As expected, the brain concentration of the BBB permeability marker (NaFL) was significantly higher in the *Lepr^db^*^/*db*^ mice (diabetic untreated group) than in the remaining groups (Figure 3B). The brain concentration of NaFL ± SEM in µg.g^−1^ is listed in Table 1. The BBB permeability of NaFL, calculated by dividing the brain concentration by the AUC, is depicted in Figure 3C. The values presented in Table 2 illustrate the increase in BBB permeability in the diabetic mice compared with the control mice. However, these observations could be affected by the small sample size.

## 3. Discussion

In this study, we evaluated the molecular and functional characteristics of the BBB in a diabetic mouse model (*Lepr^db^*^/*db*^) upon insulin administration. Our results indicated that mRNA levels of *VCAM-1*, *ICAM-1*, and *S100b* proteins were altered in *Lepr^db^*^/*db*^ mice and that some of these changes were affected by insulin treatment. Additionally, we analyzed the expression of proteins and tracked the BBB architecture using double and triple immunofluorescence labeling.

Our Western blot studies indicated that the protein expression of ZO-I was not altered, while functionality indicated a change in the BBB. This could be driven by changes in other BBB compartments. It might indicate that other markers, such as claudin-5, could be mechanistically involved in this dysregulation [32,33].

A review has highlighted the changes in the BBB in subarachnoid hemorrhage. It reported the molecular role of p53–NF-κB–MMP-9 signaling in the pathology of subarachnoid hemorrhage. Further, it indicated the involvement of tight junction proteins, including ICAM-1 and VCAM-1. These proteins were dysregulated due to increased pro-inflammatory cytokines [34]. In line with this, a previous study reported substantial BBB leakage in young *Lepr^db^*^/*db*^ mice, referred to as the pre-hyperglycemic metabolic stage, which was correlated to stress-related endothelial changes [35].

Further, in both *Lepr^db^*^/*db*^ and streptozotocin diabetic mice models, the gene level of BBB markers was altered. In this report, the regression analysis of BBB permeability examined by NaFL accumulation versus blood glucose levels in the streptozotocin-diabetic mouse model indicated a greater permeability and a strong correlation. Compared to heterozygous mice with normal blood glucose levels, *Lepr^db^*^/*db*^ mice exhibited a more than two-fold accumulation of NaFL [36]. This evidence supports the role of lost BBB integrity in diabetic animal models.

qRT-PCR identified differences in the molecular expression of the *VCAM-1*, *ICAM-1*, and *S100b* genes. There were differences in the mRNA expression of the genes in WT mice, as well as a significant difference in the expression of *VCAM-1* and *ICAM-1* in *Lepr^db^*^/*db*^ mice compared with wild-type mice. For example, *ICAM-1* expression was highest in the DB-CONT mice and lowest in DB+INS. The level was significantly reduced in *Lepr^db^*^/*db*^ mice upon treatment with insulin, indicating a mechanistic link between molecular abnormalities in the BBB and insulin signaling.

A recent study reported that insulin impacts the BBB endothelium and insulin resistance is linked to a dysfunctional BBB structure and components using RNA sequencing, functional annotation, and gene ontology [37]. In support of the role of insulin in the integrity of the BBB, our observations indicated that *ICAM-1* mRNA expression was reduced toward a normalized level compared to the wild-type control following insulin administration in *Lepr^db^*^/*db*^ mice. Further, *VCAM-1* production within *Lepr^db^*^/*db*^ mice was reduced, and insulin signifies its mRNA expression. In line with this, the mRNA expression of *S100b* was reduced in *Lepr^db^*^/*db*^ mice treated with insulin. A previous study concluded that diabetes alters the tight junctions’ molecular anatomy by affecting the structural protein content [38].

*VCAM-1* expression was highest in insulin-treated WT and *Lepr^db^*^/*db*^ mice, with the DB-CONT exhibiting the least expression. Upon treatment with insulin, the mRNA level of *VCAM*-1 in *Lepr^db^*^/*db*^ mice was reversed and significantly increased in *Lepr^db^*^/*db*^ mice. The discrepancies in the *ICAM-1* and *VCAM-1* baseline levels suggest a compensatory mechanism in the neurovascular unit in *Lepr^db^*^/*db*^ mice. Consistent with this finding, in a previous study utilizing a systemic lupus erythematosus mouse model, both markers exhibited a differential role in modulating leukocyte–endothelial interactions [39]. A postmortem study indicated that cerebrovascular expression of ICAM-1 was not altered with aging. This could be attributed to the differences in brain regions utilized. Another factor that could contribute to this observation is the existence of different medical conditions in the study subjects [40].

Our findings indicated that *ICAM-1* and *VCAM-1* expression levels presented opposite trends. In a previous study utilizing human umbilical vein endothelial cells, induction of p38 mitogen-activated protein (MAP) kinase signaling elevated the surface expression of *VCAM-1* with no impact on *ICAM-1* expression. This suggests that both adhesion molecules may have differential post-transcriptional activation [41].

Our RT-PCR analyses indicated differences in the mRNA level of BBB markers in both treated mice in the wild-type group and *Lepr^db^*^/*db*^ mice. The differences in these changes could be attributed to variations in the glucose levels of the experimental and control mice.Glucose levels in mice influence the production of ROS, and these activate different pathways that influence the expression of adhesion molecules. VCAM-1, ICAM-1, and MMP-9 are soluble adhesion molecules. ICAM-1 and VCAM-1 influence inflammatory processes in the endothelium. Their activities play a significant role in the development of atherosclerosis as they overlap with the systems that transfer leukocytes into the vascular wall. In addition, MMP-9 plays a vital role in destroying the atherosclerotic plaques building up in the extracellular matrix as they have thrombogenic properties. Importantly, atherosclerosis is a macrovascular complication of DM, and these adhesion molecules have a role to play in its development [42].

Our studies indicated that the mRNA level of *S100b* was significantly reduced in *Lepr^db^*^/*db*^ and control mice treated with insulin, suggesting a therapeutic modulation of *S100b* by insulin. S100b is a pro-inflammatory calcium-binding protein linked to inflammatory macrophages and adipocytes [43,44]. One explanation for our observation is through the Advanced glycation end-product (AGE). The AGE levels in hyperglycemia are affected, altering gene expression in cells and thereby impairing cell signaling in the extracellular environment [45]. Activation of the receptors for AGEs (RAGE) increases when glucose levels are high and under oxidative stress, elevating the production of pro-inflammatory cytokines [46]. RAGE is a multi-ligand receptor that binds to various molecules, including S100b. Therefore, there was a reduction in the production of pro-inflammatory cytokines and molecules, which, as expected, also reduced the levels of adhesion molecules [47]. This indicated the potential involvement of RAGE signaling and downstream pathways in a hyperglycemic state. Another possible explanation for this reduction in *S100b* is that insulin administration regulates adipocytes [48]. A substantial amount of S100b is contained within the adipocytes. Hormonal regulation of adipocytes via treatment with insulin has been found to modulate S100b [43]. Additionally, in streptozotocin-induced diabetes, the S100b protein was reported to be elevated by two, yet the mRNA was reduced [49]. On the other hand, exposure to elevated glucose levels was found to mediate a reduction in the protein level of S100b in neuronal cell culture. This could be driven by the disruption of cellular metabolism and energetic homeostasis [50].

In regard to the expression of ZO-1, a previous study reported that in aged mice, immunofluorescence expression of occludin-1 and ZO-1 is altered compared to young mice [51]. The endothelial cells in the BBB restrict permeability and the entry of substances into and out of the brain and CNS. The BBB has specialized characteristics, and the endothelial cells contain pericytes and proteins embedded in its capillary membrane, conferring its restrictive permeability.

In addition, functional and structural breakdowns within the BBB were observed in *Lepr^db^*^/*db*^ mice. These observations led to a cognitive deficit and macrophage infiltration [52]. Another report examined the ultrastructure of the BBB using electron microscopy. Their findings indicated that *Lepr^db^*^/*db*^ mice exhibited leakage and hyperpermeability in the BBB at younger ages [35].

Further, using two animal models of diabetes, one of them being *Lepr^db/db^* mice, BBB microvascular density was augmented while functional perfusion using fluorescein perfusing studies was not altered. This suggests that this augmentation within the structural density and increased remodeling in *Lepr^db^*^/*db*^ mice could be a non-functional compensatory mechanism [53]. In addition, in another genetic model, Desert Hedgehog-deficient mice, an impairment in the BBB was reported. Further, in *Lepr^db^*^/*db*^ mice, the reduced expression of Desert Hedgehog was linked to amplified endoneurial capillary permeability and the reduced protein expression of Claudin5 [54].

Another critical factor is that leptin receptors play a fundamental role in transferring leptin across the BBB [55]. Interestingly, the neuronal microvascular units, choroid plexus, and leptomeninges express short isoforms of leptin receptors [56]. Further, it was suggested that leptin receptors mediate leptin transcytosis via the choroid plexus [57]. This distribution supports the importance of leptin signaling in maintaining the functional integrity of the BBB.

In this study, we observed an increase in the brain concentration of NaFL in obese control mice (untreated mice) compared with all other groups and a non-significant increase in BBB permeability. The BBB specializes in reducing substances’ permeability into the brain and CNS. In a diseased state, the BBB breaks down, leading to the entry/leakage of harmful substances into the CNS, infiltration of cellular components, and aberrant transport and clearance of important molecules. The cerebral flow of blood is also affected, leading to neurological deficits. In DM, high glucose levels affect the BBB’s microvascular integrity due to oxidative stress caused by increased ROS levels and the impaired function of the antioxidant protective system. In line with our findings, a previous report indicated elevated BBB permeability in *Lepr^db^*^/*db*^ mice compared to wild-type controls and heterozygous mice [36].

### Study Limitations

This study exhibited some limitations. For example, our immunofluorescence studies demonstrated the expression of ZO-1. Yet, future studies are needed to comprehensively analyze the BBB’s subcellular features, including the intensity profiles within the endothelial cell membrane [58,59,60]. Further, our conclusion would be stronger if we characterized the ultrastructural integrity of the BBB using advanced techniques such as transmission electron microscopy (TEM). According to the human protein Atlas, ZO-1 is expressed in multiple types of neuronal cells and structures [61]. Therefore, we could examine other BBB markers. Claudin-5 has been utilized extensively in the context of BBB integrity and functionality [62,63,64,65]. Our data indicated that mRNA was altered, yet we have not confirmed that at the protein level. The notion that altered mRNA levels are linked to changes in the protein level is not strongly supported [66,67]. Thus, future studies should characterize VCAM-1, ICAM-1, and MMP-9 protein expression.

## 4. Materials and Methods

### 4.1. Animals

Adult male *Lepr^db^*^/*db*^ mice (six–eight weeks old; Strain B6.BKS (D)-*Lepr^db^*/J, Stock No. 000697 B6 db) and age-matched non-diabetic lean control mice (littermate wild-type) were included in the study. This valid diabetic model has been utilized extensively in multiple studies using the same vendor, including by our research team [68,69,70,71].

The mice were housed at the Animal Care Centre, King Saud University, and had free access to food and water. All experiments were performed in compliance with the rules of the Research Ethics Committee (REC) at King Saud University with ethics reference number (SE-19-135); the date of issue is 13-2-2020.

Mice were bred in the animal care facility by mating heterozygous (*Lepr^db^/^+^*) males and females. Mice genotypes were confirmed by tail tissue and PCR analysis conducted by Transnetyx, Inc. (Cordova, TN, USA). The mice were maintained at a room temperature of 22 ± 2 °C with a 12:12 h light/dark cycle and 40 to 60% humidity.

### 4.2. Experimental Design

Four groups of mice were treated intraperitoneally (IP) with either normal saline (control) or insulin (0.1–0.4 U/kg in normal saline, a unified dose was given to the groups at the same time), as follows [72]:

Group I WT (wild-type mice): 6–8 mice were treated with normal saline for two weeks.

Group II *Lepr^db^*^/*db*^ (*Lepr^db^*^/*db*^ mice): 6–8 mice were treated with normal saline for two weeks.

Group III WT—insulin-treated (wild-type insulin-treated mice): 6–8 mice received insulin daily for two weeks.

Group IV *Lepr^db^*^/*db*^—insulin-treated (*Lepr^db^*^/*db*^ insulin-treated mice): 6–8 mice were treated with insulin daily for two weeks.

The insulin source was HUMALOG^®^ Mix50/50^TM^ (Eli Lilly and Company, Indianapolis, IN, USA, HP8798). To confirm the presence of diabetes in the models, the mice fasted for 4–6 h. Their blood glucose levels were then measured using 1–2 drops of blood collected from their tails on a glucometer chip (Accu-Check Advantage Blood Glucose Monitor, Roche Diagnostics Corp., Indianapolis, IN, USA). All *Lepr^db^*^/*db*^ mice had their blood sugar levels tested every two days. The control group had less than 200 mg/dL. The mice’s total body weight and blood glucose levels were measured regularly to assess their overall health status [73,74].

### 4.3. Tissue Collection

Mice were anesthetized with isoflurane (4% for induction, then 1–1.5% for maintenance) in 30% O_2_ using a facemask and SurgiVet vaporizer (Sigma-Aldrich, Saint Louis, MO, USA). A toe pinch was applied beforehand to ensure the animal was deeply anesthetized. The anesthesia dose increased if the animal responded to the toe pinch. The mice were then decapitated, and their brains were immersed immediately in liquid nitrogen and kept at −80 °C for further processing [73].

### 4.4. BBB Permeability Experiment

#### 4.4.1. Pharmacokinetic Studies

The functional integrity of the BBB in *Lepr^db^*^/*db*^ mice (one hemisphere of the brain) was examined using sodium fluorescein (NaFL) analysis, a low-molecular-weight hydrophilic compound [42,75]. The solution of 5 mg/mL Na fluorescein was prepared by dissolving 50 mg of Na fluorescein in 10 mL of sterile saline, following which the pH was checked and was found to be 7.4. Mice were anesthetized with isoflurane (4% for induction, then 1–1.5% for maintenance) in 30% O_2_ using a facemask and SurgiVet vaporizer. When the mice were completely anesthetized, both jugular veins were exposed. They were then injected with doses of 20 mg/kg per mouse in the left jugular vein. Blood samples were collected after 1, 5, 10, 20, and 30 min from the contralateral jugular vein. Following the collection of the last blood sample (30 min), the blood was washed out through the left ventricle (LV) of the heart with PBS + 10 units of heparin (this is known as the trans-cardiac buffer washout method). Plasma samples were separated from the blood by centrifuging for 10 min with 5000× *g* at 4 °C. Aliquots (10 µL) of total plasma samples (bounded fraction of NaFL) were diluted up to 1000 µL (1:100) with a solution of 4% bovine serum albumin in water [76].

#### 4.4.2. Spectrofluorometer Analysis of Na+ Fluorescein Permeability in the BBB

The concentration of NaFL in plasma and brain samples was measured using a Fluostar Optima spectrofluorometer (BMG, LabTech). The standard curve (62.5, 125, 250, 500, 1000, 2500, and 5000) of NaFL was prepared from a serial dilution of NaFL in the plasma and brain matrix. Detection of the fluorescence was performed at excitation and emission wavelengths of 485 and 520 nm, respectively. A total of 20 µL of each plasma and brain sample was added to a 96-well plate for analysis [77,78].

#### 4.4.3. Pharmacokinetic Analysis

The areas under the plasma concentration–time curve (AUC) of NaFL during the sampling time (0–30 min) were estimated using the linear trapezoidal rule. The apparent brain uptake clearance ($K_{in}$) for NaFL was calculated by dividing the brain concentration of NaFL (NaFL ${Br}_{Conc}$) at 30 min by ${AUC}_{0-30}$ of NaFL in plasma (0–30 min) ($K_{in}={Br}_{Conc}/{AUC}_{0–30}$ (Equation (1))) [79].

(1) $$K_{in}=\frac{{Br}_{Conc}\;}{{AUC}_{0–30}}$$

### 4.5. Quantification of mRNA Expression by Real-Time Polymerase Chain Reaction

The molecular expression of BBB markers was examined in the brain homogenate of *Lepr^db^*^/*db*^ mice. Quantitative analysis of specific mRNA expression was performed using RT-PCR by subjecting the resulting cDNA to PCR amplification using 96-well optical reaction plates in the ABI Prism 7500 Fast real-time PCR System (Applied Biosystems, Waltham, MA, USA). The 25 µL reaction mixture contained 0.1 µL of a 10 µM forward primer and 0.1 µL of a 10 µM reverse primer (40 nM final concentration of each primer), 12.5 µL of SYBR Green Universal Master mix, 11.05 µL of nuclease-free water, and 1.25 µL of the cDNA sample. Mouse primers and probes for *ICAM-1*, *VCAM-1*, *MMP9*, *S100b*, and *GAPDH* were utilized (Table 3). The primers were purified using standard desalting and purchased from Integrated DNA Technologies (IDT, Coralville, IA, USA). The RT-PCR data were analyzed using the relative gene expression (i.e., ΔΔ CT) method, as described and explained previously [80]. The data are presented as the fold change in gene expression normalized to the endogenous reference gene GAPDH and relative to a calibrator. The fold change in the level of target genes between treated and untreated groups was corrected by the level of GAPDH. After treatment for the periods specified, total cellular RNA was isolated using TRI-zol reagent (Invitrogen^®^) following the manufacturer’s instructions.

#### 4.5.1. CDNA Synthesis

First-strand cDNA synthesis was performed using the High-capacity cDNA reverse transcription kit or iScript RT supermix (Thermo Fisher Scientific, Waltham, MA, USA, catalog# 4368814). Briefly, 1.5 µg of total RNA from each sample was added to a mixture of 2.0 µL of 10× reverse transcriptase buffer, 0.8 µL of 25× dNTP mix (100 mM), 2.0 µL of 10× reverse transcriptase random primers, 1.0 µL of MultiScribe reverse transcriptase, and 3.2 µL of nuclease-free water. The final reaction mixture was then kept at 25 °C for 10 min, heated to 37 °C for 120 min, heated further to 85 °C for 5 min, and then cooled to 4 °C.

#### 4.5.2. Real-Time Polymerase Chain Reaction (RT-PCR)

RT-PCR was conducted on a liquid nitrogen snap-frozen prefrontal cortex (PFC), a brain region linked to cognitive capacity, and the PFC was microdissected on a cryostat (Leica CM3050 S cryostat—Leica Microsystems). Total RNA was isolated using a Trizol reagent following the manufacturer’s instructions (Invitrogen Co., Grand Island, NY, USA). The Nanodrop (NanoDrop™ 8000 spectrophotometer, Thermo Fisher Scientific, Waltham, MA, USA) was used to test the purity of the RNA samples. Our sample readout, measured in concentration of ng/μL, ranged between 1.8 to 2. iScript RT supermix (Bio-Rad Laboratories, catalog# 1708840) was then used to reverse the total mRNA transcribed to cDNA, which was then amplified with the selected primers: VCAM-1, ICAM-1, MMP-9, and S100b (genes involved in BBB structure and function). The levels of transcription of the primers were normalized with suitable housekeeping genes. The real-time PCR analyses were performed using the CFX96 Real-Time Detection System (Bio-Rad) [81].

### 4.6. Immunofluorescence

Immunofluorescence, confocal imaging, and image analysis were performed to track and analyze the architecture of BBB markers in the prefrontal area slices of *Lepr^db^*^/*db*^ and control mice [73]. Frozen mouse brain tissue was sectioned (14–20 µm thickness) using a cryostat (Leica CM3050s). Brain sections were washed with 1% PBS, pre-incubated with 4% paraformaldehyde for 30 min, and incubated with a permeabilizing agent (1% Triton, 0.5% Tween in PBS, or −20 °C acetone). Slices were then washed extensively with 1% PBS and incubated with 10% normal goat serum NGS (Sigma-Aldrich) in 1% TBS containing 0.3% Triton X-100 for one hour. This was followed by overnight incubation at 4 °C with rabbit anti-ZO-1 (1:300, Novus cat no# NBP1-85047) and anti-occludin (1:500, Novus cat no# NBP1-77037ss) primary antibody in 3% bovine serum albumin (BSA; Sigma-Aldrich) and 1% PBS containing 0.1% Tween-20. The following day, sections were washed with 1% PBS and incubated with appropriate secondary antibodies (1:250, Invitrogen) for one hour in a 1% PBS solution containing 3% BSA and 0.1% Tween-20. After incubation, the tissue was washed five more times with 1% PBS or TBS buffer solution. Finally, glass slides were placed in an oven at 30–32 °C for 10–15 min to dry and then coverslipped using Fisherfinest^®^ Premium Cover Glass (Fisher Scientific) with ProLong^®^ Gold anti-fade or ProLong^®^ Gold anti-fade mountant with Dapi reagents (Life Technologies, catalog number P36941) [73].

### 4.7. Confocal Microscopy

All confocal images were obtained on a Zeiss LSM-800 confocal microscope with a Fluar (5×/0.25) objective, a Plan-Apochromat (20×/0.75 na) objective, a C-Apochromat (40×/1.2 W Corr) objective, and a Plan-Apochromat (63×/1.46 Oil) objective. A comparison of WT and *Lepr^db^*^/*db*^ mice tissue was performed on slices prepared in parallel, and images were obtained using identical exposure times. Multitrack acquisition was performed with excitation lines at 488 nm for Alexa 488, 543 nm for Alexa 568, and 633 nm for A647. Z-series stack confocal images were taken at fixed intervals: 0.6 µm for 40× and 0.4 µm for 63×, with the same pinhole setting for all three channels. The frame size was either 1024 × 1024 or 512 × 512 pixels. Images were processed using ImageJ 1.53u U. S. National Institutes of Health, Bethesda, MD, USA, (http://imagej.nih.gov/ij) (https://imagej.nih.gov/ij/) (accessed on 4 August 2019). The contrast and brightness of the displayed images were adjusted using ImageJ. Image stacks for double immunostained slides were converted into the same single average Z-axis projection and exported as TIFF files [73].

### 4.8. Western Blotting

We analyzed the protein expression of ZO-1 in the brain homogenate of the tested groups. First, 50 mg of PFC from the brain tissues (n = 3 mice per group) were homogenized in 0.32 M sucrose solution in the presence of protease and phosphatase inhibitors. The protein concentrations of samples from the brain tissue homogenates of the tested groups were measured using a NanoDrop™ 8000 Spectrophotometer (Thermo Fisher Scientific, Waltham, MA, USA), mixed with 2X lamellae buffer, denatured for 10 min at 95 °C, and separated on 7.5% SDS-PAGE gel. The protein was electrophoretically transferred from the gel into a nitrocellulose membrane. Membranes were blocked with 5% nonfat dry milk in TBS-T for 30 min and probed with the primary antibody, Rabbit anti-TJP-1(1:500, Novus), TJP-1 represents ZO-1, in a blocking solution. They were then treated with horseradish-peroxidase conjugated secondary antibodies and ECL Western blotting detection reagents. The signals were detected and measured with a luminescent image analyzer (ChemiDoc TMMP Bio-Rad). ImageJ was used to measure the intensity of ZO-1 [82].

### 4.9. Statistical Analysis

The data were reported as the mean ± standard error of the mean (SEM). For our studies, differences between the groups were determined using a two-way ANOVA (treatment × Genotype) followed by a Tukey–Kramer post-hoc test. GraphPad Prism software Version 10 (GraphPad Software, San Diego, CA, USA) was used to perform the calculations, and differences were considered significant at *p* < 0.05.

## 5. Conclusions

This study evaluated the functional alterations in diabetic mice models treated with insulin and controls that received saline treatments. The expression of significant proteins of the BBB, such as ZO-1 and occludin, was also evaluated; these results indicated that insulin injections reversed the effects of diabetes and hyperglycemia in the mice and exerted positive effects on the BBB. The expression of adhesion molecules was altered. Moreover, the brain concentration of NaFL, along with the molecular change in obese mice, provides evidence for the effect of hyperglycemia on the BBB. Permeability in the obese insulin-treated mice was lower than in the saline-treated mice, although this difference was not significant. This indicates the need for further studies investigating the effects of insulin treatment on the permeability of the BBB and how hyperglycemic conditions promote reduced integrity. Future work will focus on the architecture of other brain regions or proteins in the BBB. In addition, a behavioral study will be conducted to ensure the effect of insulin treatment on cognition from changes in the BBB, and more research will be needed to examine the most common treatments used in diabetic patients with high permeability and their toxicity on the patient due to molecular and functional changes in the BBB.

## Figures and Tables

**Table 1:** The brain concentration of NaFL.

Groups (n = 3)	Mean	Std. Error of Mean
**WT**	1.204	±0.1479
**WT Treated**	1.613	±0.02765
*Lepr^db^* ^/*db*^	2.044 **	±0.01843
*Lepr^db^*^/*db*^ **Treated**	1.406	±0.1607

Examination of the brain concentration using the NaFL marker. Data represent mean ± S.E.M. Statistical differences were assessed using a two-way ANOVA. ** *p* < 0.01. Groups are Wild-type control = WT; Wild-type Treated with Insulin = WT Treated; *Lepr^db^*^/*db*^ Control = *Lepr^db^*^/*db*^; and *Lepr^db^*^/*db*^ treated with insulin = *Lepr^db^*^/*db*^ treated.

**Table 2:** The BBB permeability of NaFL.

Groups (n = 3)	Mean	Std. Error of Mean
WT	0.5210	±0.05255
WT Treated	0.6077	±0.04840
*Lepr^db^* ^/*db*^	0.7883	±0.05990
*Lepr^db^*^/*db*^ Treated	0.5433	±0.06972

Examination of BBB permeability of NaFL. Data represent mean ± S.E.M. Statistical differences were assessed using a two-way ANOVA. Groups are Wild-type control = WT; Wild-type Treated with Insulin = WT Treated; *Lepr^db^*^/*db*^ Control = *Lepr^db^*^/*db*^; and *Lepr^db^*^/*db*^ treated with insulin = *Lepr^db^*^/*db*^ treated.

**Table 3:** Primer Sequences.

Gene	Name	NCBI ID	Forward	Reverse
*ICAM-1*	intercellular adhesion molecule 1	NM_010493.3	GTG ATG GCA GCC TCT TAT GT	GGG CTT GTC CCT TGA GTT T
*VCAM-1*	vascular cell adhesion molecule 1	NM_011693.3	GAG GGA GAC ACC GTC ATT ATC	CGA GCC ATC CAC AGA CTT TA
*MMP*	matrix metallopeptidase 9	NM_013599.4	TGC ACT GGG CTT AGA TCA TTC	TGC CGT CTA TGT CGT CTT TAT TC
*S100b*	beta polypeptide, neural	NM_009115.3	GCA AAT GAT GCT CCA GAA AGT AG	TGC ACA CTG TGG AAG AAG AG
*Gapdh*	Glyceraldehyde-3-phosphate dehydrogenase isoform 1	NM_001289726.1	GTGGCAAAGTGGAGATTGTTG	CGTTGAATTTGCCGTGAGTG

## Data Availability

Data are available upon reasonable request.

## Supplementary Materials

The following supporting information can be downloaded at: https://www.mdpi.com/article/10.3390/ijms25147768/s1, Right panels represent high magnification of the white boxes in (A). Scale bars represent 35 μm.

## Abbreviations

BBB	Blood-Brain Barrier
DM	Diabetes Mellitus
T1DM	Type 1 Diabetes Mellitus
T1DM	Type 1 Diabetes Mellitus
ICAM	Intercellular Adhesion Molecule
MMP	Matrix Metalloproteinases
NaFL	Sodium Fluorescein
RT-PCR	Real-Time Polymerase Chain Reaction
VCAM	Vascular cell adhesion protein
S100b	S100 calcium-binding protein B
*Lepr* ^*db*/*db*^	Leptin receptor mutant mice
TJP-1	Tight junction protein 1 (Zonula occludens-1)
ZO-1	Zonula occludens-1
