# Supplementary material for: Increased Permeability of the Blood–Brain Barrier in a Diabetic Mouse Model (Leprdb/db Mice)
Source: Int J Mol Sci. 2024 Jul 16;25(14):7768. doi: 10.3390/ijms25147768 (PMC11276738; doi:10.3390/ijms25147768)
Supplement: Supplementary file 1 [file ijms-25-07768-s001.zip › ijms-3041218-supplementary.pdf]

# Increased Permeability of the Blood-Brain Barrier in Diabetic Mouse Model (*Lepr*$^{db/db}$ mice)-Supplementary.

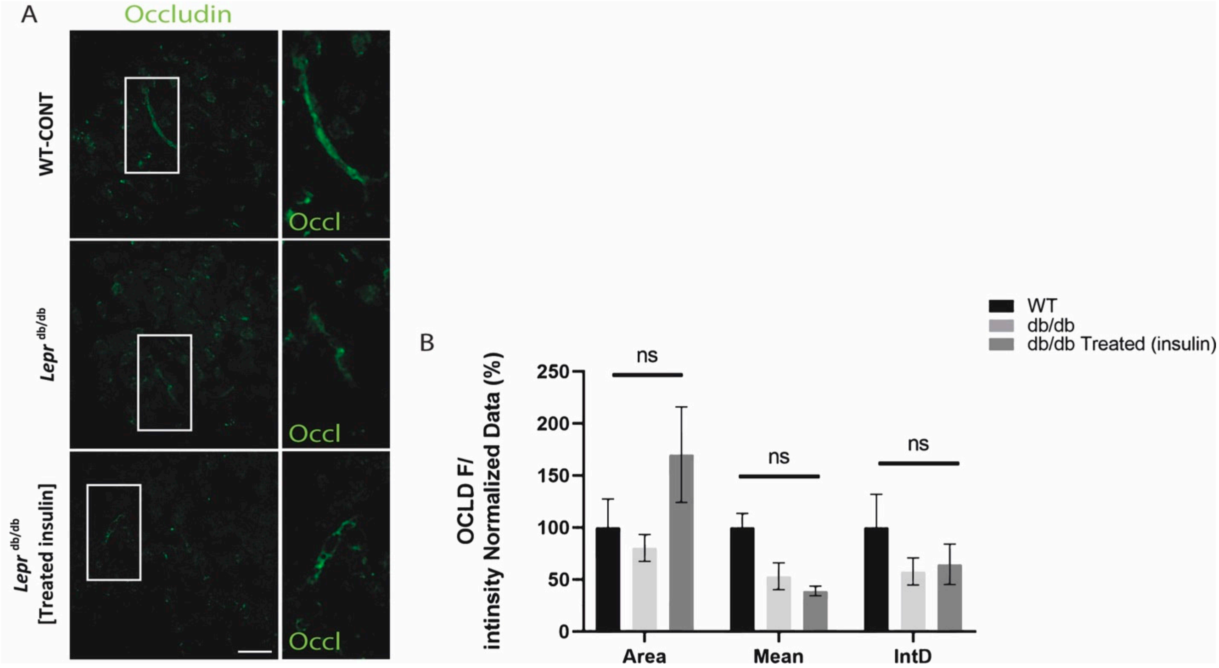


Supplementary Figure S1. (A) The green channel represents confocal images of occludin immunofluorescence in wild-type and *Lepr*$^{db/db}$ mice. (B) Total quantifications of occludin expression. n=3 mice per group.
